# Extracellular Vesicles as Biomarkers of Pregnancy Complications

**DOI:** 10.3390/ijms252211944

**Published:** 2024-11-06

**Authors:** Anastasiia K. Popova, Elena S. Vashukova, Roman A. Illarionov, Anastasia R. Maltseva, Olga V. Pachuliia, Tatiana B. Postnikova, Andrey S. Glotov

**Affiliations:** 1 Department of Genomic Medicine, D.O. Ott Research Institute for Obstetrics, Gynecology, and Reproduction, St. Petersburg 199034, Russia; 2 Department of Genetics and Biotechnology, St. Petersburg State University, St. Petersburg 199034, Russia

**Keywords:** extracellular vesicles, pregnancy, pregnancy complications, biomarkers

## Abstract

Extracellular vesicles (EVs) are double-membrane vesicles that facilitate intercellular communication and play a pivotal role in both physiological and pathological processes. A substantial body of evidence suggests that EVs play a role in the pathogenesis of various pregnancy complications. Because EVs can be detected in the peripheral blood, they are potential biomarkers for the early diagnosis of pregnancy complications and foetal developmental disorders. The majority of studies have demonstrated a correlation between alterations in the concentration of EVs and changes in their contents and the occurrence of pregnancy complications. Despite the current limitations in establishing a clear link between these findings and the pathogenesis of the disease, as well as the lack of sufficient evidence to support their use in clinical practice, it is noteworthy to highlight the potential role of specific miRNAs carried by EVs in the development of pregnancy complications. These include miR-210 and miR-136-5p for pre-eclampsia and gestational diabetes mellitus, miR-155, miR-26b-5p, miR-181a-5p, miR-495 and miR-374c for pre-eclampsia and preterm birth. The following miRNAs have been identified as potential biomarkers for preterm birth and gestational diabetes mellitus: miR-197-3p and miR-520h, miR-1323, miR-342-3p, miR-132-3p, miR-182-3p, miR-517-3p, miR-222-3p, miR-16-5p and miR-126-3p. Additionally, miR-127-3p has been linked to foetal growth restriction and preterm birth. Nevertheless, it would be premature to propose that EVs can be employed as biomarkers for pregnancy complications. Further research and the accumulation of results obtained using the methods proposed in the MISEV2023 guidelines will enable a definitive conclusion to be reached.

## 1. Introduction

Pregnancy complications remain a significant concern in modern obstetrics and gynaecology, accounting for a considerable proportion of maternal and child morbidity and mortality [1,2,3]. Despite the extensive history of research, the aetiology and pathogenesis of the majority of pregnancy complications remain poorly understood, and accurate diagnostic methods are lacking [2]. In recent years, extracellular vesicles (EVs), which facilitate intercellular communication by transferring biologically active substances and regulate various cellular processes, have emerged as a subject of interest for researchers [2].

Extracellular vesicles (EVs) are nano- and micro-sized membrane vesicles secreted by cells into the extracellular space [4,5]. They serve as carriers of a variety of biological molecules, including proteins, lipids, DNA fragments, and coding and non-coding RNAs [4,6]. Among these, microribonucleic acids (microRNAs, miRNAs) and circular RNAs (single-stranded RNAs covalently closed in a continuous loop) play a distinctive role, primarily regulating gene expression at the transcription level [7].

It has been demonstrated that EVs are implicated in a range of physiological processes, including intercellular communication, blood coagulation, angiogenesis, inflammation, immune responses and reparative reactions [5]. Additionally, EVs have been demonstrated to participate in the advancement of diverse pathological processes. The available evidence indicates that EVs have the potential to serve as a diagnostic and monitoring tool for a range of diseases, including cancer, cardiometabolic, neurological, respiratory, dermatological, liver and kidney diseases, and for the detection of transplant rejection [4,5,6]. The principal advantage of utilising EVs is the capacity to obtain them from a multitude of biological fluids (plasma, serum, urine, saliva, breast milk, amniotic fluid, and others), which minimises inconvenience for patients, accelerates analysis, and reduces financial costs [6,8]. Conversely, EVs have a tissue-specific origin and reflect the functional status of the original cells, which underscores the utility of their use as biomarkers of various diseases.

During pregnancy, EVs facilitate foetal-maternal communication and are involved in numerous physiological processes, including embryo implantation, trophoblast migration and invasion, immunomodulation, cellular adaptation to physiological changes, spiral artery remodelling and uterine preparation for delivery [9,10]. A substantial body of research has been conducted to examine the correlation between alterations in the concentration of circulating EVs and their contents and the emergence of diverse pregnancy complications [2,11,12,13,14,15].

This review presents an overview of the current data on the involvement of EVs in the pathogenesis of pregnancy complications, including pre-eclampsia (PE), gestational diabetes mellitus (GDM), foetal growth restriction (FGR) and preterm birth (PTB). It considers the possibility of utilising EVs as biomarkers for these pathological conditions.

A literature search was conducted in the Scopus and PubMed databases using the following keywords: extracellular vesicles, pregnancy, pregnancy complications, preeclampsia, gestational diabetes mellitus, foetal growth restriction, preterm labour, exosomes, microparticles, placental extracellular vesicles. The period under review encompasses studies published between 2013 and 2024. The findings of experimental studies and review articles were subjected to analysis.

## 2. Extracellular Vesicles: Characteristics, Types and Methods Analysis

The classification of EVs is dependent on a number of factors, including size, mode of formation and function. The three main types of EVs are exosomes, ectosomes or microparticles (microvesicles) and apoptotic bodies [14].

The most extensively studied are exosomes, which are the smallest EVs, with diameters ranging from 30 to 150 nm [4]. Exosomes are produced by late endosomes, which are also known as multivesicular bodies (MVBs) [16]. The invagination of the endosomal membrane results in the formation of intraluminal vesicles within the MVBs [16]. MVBs can either undergo fusion with lysosomes, resulting in degradation, or move towards the plasma membrane [16]. Following the fusion of MVBs with the cell membrane, the intraluminal vesicles are released into the extracellular space, where they form exosomes [17]. Proteins of the endosomal sorting complex required for transport (ESCRT) and related proteins (Alix, TSG101, HSC70 and HSP90β), as well as cytoskeleton proteins (actin, syntenin and moesin), signal transduction proteins (kinases) and metabolic enzymes (GAPDH, LDHA, PGK1, aldolase and pyruvate kinase) have been identified within exosomes [6].

Microvesicles are formed by the outward budding of the plasma membrane with the participation of cytoskeleton filaments [18]. The diameters of these particles range from 100 to 1000 nm [19]. Microvesicles contain a variety of proteins, including cytosolic and plasma membrane-associated proteins (tetraspanins), cytoskeleton proteins, heat shock proteins, integrins and proteins with post-translational modifications, such as glycosylation and phosphorylation [6,20].

Apoptotic bodies exhibit a range of sizes, from 50 nm to 5000 nm, and are formed as a consequence of programmed cell death (apoptosis) [21]. This process involves the dynamic protrusion of the plasma membrane and subsequent fragmentation into individual apoptotic bodies [21]. The contents of apoptotic bodies include cellular components such as intact organelles, chromatin, fragmented DNA, micronuclei, cytoplasmic contents and degraded proteins [21]. The protein profile of apoptotic bodies is analogous to that of cell lysate, exhibiting elevated levels of proteins associated with the nucleus (histones), mitochondria (HSP60), the Golgi apparatus and the endoplasmic reticulum (GRP78) [6]. 

The classification of EVs into exosomes, microvesicles and apoptotic bodies is currently a topic of contention within the scientific community. The functional characteristics of different groups of EVs formed by the same cells may be similar, and determining the precise biogenesis of a specific particle is a challenging undertaking. In light of these considerations, the International Society for Extracellular Vesicles (ISEV) proposes the use of the overarching term “extracellular vesicles” [22].

A variety of molecular biology techniques are employed to examine EVs, which furnish data regarding particle dimensions, their distribution in biological fluids, the specific molecules present on their surface, and the contents they carry [6,19,23]. Detailed descriptions and illustrations of all methods are presented in Minimal information for studies of extracellular vesicles (MISEV2023) [24].

The separation of EVs from multicomponent biological fluids (plasma, urine, etc.) represents a significant challenge [24]. Since biological fluids have a complex composition that contains various components that are similar in size to EVs: proteins, protein complexes, lipoproteins of different densities, membrane vesicles of different genesis, and non-vesicular membrane formations [24]. At present, a number of methods for the isolation of EVs are employed in laboratory practice. However, these methods lack standardisation and are associated with a number of disadvantages. The selection of an appropriate method is dependent upon the specific objectives of the experiment and the capabilities of the experimenter. The problem is that the scientific results obtained and published depend on the method chosen by the researchers and are often not comparable. In 2024, the International Society for Extracellular Vesicles (ISEV) published the ‘Minimum Information for Studies of Extracellular Vesicles’ (MISEV2023) guidelines, which presented the available methods for obtaining and characterising EVs along with their respective advantages and limitations [24]. This document is of significant assistance to researchers in the selection of appropriate methods, thereby contributing to the rigour, reproducibility and transparency of EVs research. It ensures that conclusions are supported by the experiments performed and the information provided, thus guaranteeing the reliability of the findings.

The conventional techniques employed for the isolation of EVs are founded upon the distinctive attributes of these vesicles. For example, differential centrifugation, ultracentrifugation, ultrafiltration and gel filtration are methods based on the size and physical density of EVs [25,26,27]. However, EV preparations isolated by these methods are typically contaminated with components of biological fluids of a similar size and density. Immunoaffinity methods are based on the interaction between antibodies and protein molecules that are integral to the composition of the vesicular membrane [28]. These methods are employed for the purpose of obtaining EVs from specific cell types [28]. Furthermore, these methods are employed for the isolation of a particular type of EVs, such as exosomes, from a heterogeneous mixture of EVs [29].

Precipitation is the simplest but most effective method to isolate EVs with minimal loss, but the sample will contain many impurities [24]. The ultracentrifugation method is regarded as the benchmark against which the efficacy of any novel technology is typically evaluated [25]. The principal advantage of this method is the possibility of standardisation. However, it is also notable for its labour intensity and high cost of equipment. The ultrafiltration method offers the advantage of being readily manufacturable, requiring neither specialised equipment nor qualifications and being relatively labour-intensive. However, components of biological fluid of a non-vesicular nature with a size equal to or smaller than EVs remain in the filtrate [25]. In order to perform exclusion chromatography, it is necessary to prepare the samples for the procedure by pre-cleaning them of large impurities [30]. Additionally, there are commercially available kits for exosome isolation, including the exoEasy kit from Qiagen (Valencia, CA, USA), the ExoQuick kit from System Biosciences (Mountain View, CA, USA), the ExoMir kit from Bioo Scientific (Austin, TX, USA), the Exo-spin kit from Cell Guidance Systems (Cambridge, UK) and the Exo-Flow kit from System Biosciences (Mountain View, CA, USA) [31]. It is noteworthy that numerous kits designed for the purification of exosomes employ precipitation techniques, which result in the isolation of a mixture of EVs rather than pure exosomes. Additionally, these kits have the potential to precipitate numerous free proteins, leading to the contamination of the final preparation [24].

Information regarding the structure and biodiversity of EVs is obtained through the utilisation of imaging techniques [2]. The use of immunoelectron microscopy enables the direct detection of specific markers on the surface of nanovesicles, allowing for the visualisation of all types of EVs [32,33]. For instance, specific markers such as the tetraspanins CD81, CD9 and CD63 are employed for the visualisation of exosomes, whereas the proteins CD31, CD51 and CD105 are utilised for microvesicles [22]. Nanoparticle trajectory analysis (NTA) is based on the detection of light scattering from particle interactions and their Brownian motion [8]. The scattered light is collected by a microscope with a camera positioned at the top to capture the particle motion on video [8]. The video is then analysed by software to estimate the particle size and concentration [8].

Flow cytometry is employed for the detection and characterisation of EVs [11]. Nevertheless, this approach is appropriate for EVs with a larger diameter (300–500 nm) [34]. The investigation of smaller-sized EVs employs the use of fluorescent dyes embedded in membranes and instruments that have been adapted for the detection of nanoparticles [35]. The presence of protein complexes in body fluids with analogous biophysical characteristics to EVs may compromise the precision of the analytical process [36].

The contents of EVs are subjected to analysis using modern molecular genetic techniques. For instance, the RNA present within EVs is examined using microarray techniques, RT-PCR, ddPCR and NGS (next generation sequencing) [4]. The proteomic profile of EVs is investigated using mass spectrometry, immunoblotting, and liquid chromatography methods [4,6].

Thus, EVs are bimembrane vesicles secreted by various cell types, present in almost all body fluids and carrying a wide range of contents, including proteins, RNA and lipids, to transmit signals to specific target cells. This makes them important participants in the regulation of physiological and pathological processes. EVs display a tissue-specific pattern and reflect the functional status of the original cells, indicating their potential for use as indicators of cellular function and biomarkers of various diseases. A plethora of methodologies have been devised for the isolation and identification of EVs. In 2024, the International Society for Extracellular Vesicles (ISEV) published guidelines for research on EVs, outlining methods for isolating and analysing EVs and their contents. The scientific community was able to reach a consensus on the listed methods. This is a significant breakthrough that promises a better understanding of the functions of EVs as well as the possibility of applying EVs in clinical practice [24].

## 3. Placental Extracellular Vesicles

In addition to the pregnant woman’s own cells, the placenta is another source of EVs during pregnancy. EVs released from the placenta, known as placental extracellular vesicles (PEVs), include exosomes, microvesicles, and apoptotic bodies. These enter the maternal bloodstream primarily through the syncytiotrophoblast [8]. The molecular marker of PEVs is the placenta-specific enzyme placental alkaline phosphatase (PLAP) [8]. PEVs are vital for the interaction between the placenta and the endometrial decidual membrane, and they also regulate the maternal immune response [14,32].

PEVs can be identified in maternal blood as early as the sixth week of gestation [33]. As gestation advances, the quantity and concentration of PEVs increase [14]. Additionally, the quantity, composition and biological functions of PEVs exhibit considerable variation across the trimesters of pregnancy and in relation to placental status [2,13].

Placental exosomes have been observed to carry a number of major immunomodulatory proteins on their surface, including human leukocyte antigen (HLA)-G5, B7-H1 and B7-H3, in both early and full-term placental explants [34]. The expression of different HLA types during pregnancy is of critical importance for the development of immune tolerance [34]. Indeed, some obstetric complications are characterised by an abnormal expression of HLA molecules [34]. Syncytiotrophoblast-derived EVs have been observed to carry ligands of the human NK cell activating receptor NKG2D. These BBs have the capacity to induce immune suppression by interacting with the NKG2D receptor on NK cells, CD8 T cells, and γδ T cells, thereby reducing their cytotoxic function in vitro without altering the perforin content [37,38]. This effect of trophoblastic EVs may elucidate the mechanisms underlying the placenta’s defence against attack by maternal cytotoxic leukocytes [38].

The second trimester of pregnancy is characterised by the presence of high levels of immunosuppressive proteins, specifically transforming growth factor beta 1 (TGF-β1) and interleukin 10 (IL-10), within placental microvesicles [35]. These proteins exert a significant influence on the immune system, acting as crucial modulators [35]. Microvesicles isolated in the third trimester of pregnancy, originating from the syncytiotrophoblast, bind to B cells and monocytes, modulating the expression of certain cytokines involved in type 2 immunity and increasing the expression of IL-6 and TNF-α relative to other cytokines [36]. 

EVs of placental origin contain a variety of microRNAs (miRNAs) that can influence the proliferation, differentiation, migration, invasion, and apoptosis of trophoblast cells, as well as the functions of endothelial cells, the processes of angiogenesis, placentation, the expression of inflammatory factors and components of the renin-angiotensin system, the regulation of insulin signalling pathways, oxidative stress, and other biological processes [39,40]. A considerable number of miRNAs present in human placental extracellular vesicles (PEVs) that can be identified in maternal plasma are associated with a miRNA cluster on chromosome 19 (C19MC) [41]. MiR-517a, which is situated within this cluster, has been demonstrated to regulate tumour necrosis factor-mediated signalling [42]. Another member of this cluster, miR-519c, has been demonstrated to suppress phosphodiesterase 3B, a known stimulator of TNF-α, and may serve as a potential biomarker of preterm labour due to endotoxin-producing pathogens [43]. MiR-519d, a member of the C19MC cluster, has been demonstrated to regulate critical trophoblast functions, stimulating proliferation while inhibiting migration [44]. In hypoxic conditions, the expression of miR-1273d, miR-4492 and miR-4417 is increased in human trophoblast cells, which are involved in the regulation of immune and inflammatory processes by affecting HLA-G [45]. A recent report indicated that fragments of 5′-transport RNA (tRF) constitute the majority of small RNAs in syncytiotrophoblast EVs (STBEVs) in PE and normotensive pregnancies [46]. These molecules induce an inflammatory response in tissue macrophages, reduce endothelial nitric oxide synthase (eNOS) levels in endothelial cells, and thus may contribute to the pathophysiological changes associated with PE [46].

PEVs are involved in the majority of processes that are crucial for the successful progression of pregnancy. Consequently, alterations in the concentration, composition and biological activity of PEVs may serve as an indicator of placental dysfunction, impaired foetal-maternal communication and the emergence of pregnancy complications [14]. Furthermore, PEVs are regarded as a promising biomarker for these pregnancy pathologies, given that alterations in their secretion in the blood of a pregnant woman can be observed as early as the first trimester, preceding the onset of clinical symptoms. Additionally, sufficient quantities of biomaterial for research can be obtained non-invasively [1,2].

## 4. Extracellular Vesicles and Pregnancy Complications

From 1998 to 2021, 71 original experimental studies from a range of countries have been published, demonstrating an association between EVs and a number of pregnancy-related complications, including PE, GDM, FGR and PTB (see Table 1, Table 2, Table 3 and Table 4). Additionally, several reviews have been published on these complications of pregnancy [2,13,17,47]. Nevertheless, no meta-analyses of the data could be identified, which may be attributed to the disparate study designs.

### 4.1. Preeclampsia

Preeclampsia (PE) is a severe pathological condition that occurs after the 20th week of pregnancy. It is characterised by arterial hypertension, proteinuria and oedema [48,49]. PE complicates approximately every fifth to sixth pregnancy and is a common cause of maternal and child morbidity and mortality [48].

It is hypothesised that the development of PE occurs in two stages. At the initial stage, the interaction between the processes of trophoblast invasion into the uterine wall and remodelling of spiral uterine arteries is disrupted, which results in the abnormal formation of the placenta [50]. The second stage is typified by the introduction of placental factors into the maternal circulation, which precipitates a systemic inflammatory response syndrome and generalised endothelial dysfunction, ultimately resulting in multi-organ failure [50].

The precise relationship between abnormal placentation and inflammatory syndrome remains unclear. It is hypothesised that the development of the second phase of PE may be a consequence of pre-existing pathologies in the pregnant woman. Concurrently, a hypothesis has been proposed that PEVs may represent a pivotal determinant of the second phase of PE. Proteins present in PEVs have been identified as endogenous molecules that may contribute to their pro-inflammatory nature, including extracellular free actins, tubulins, and heat shock proteins [51]. In addition to protein components, EVs have been demonstrated to contribute to the development of PE through the transfer of miRNAs [12,52].

The first evidence of alterations in EVs content in pregnancies complicated by preeclampsia was reported in 1998 [53]. This study demonstrated that EVs levels derived from the syncytiotrophoblast were elevated in the third trimester in the plasma of patients with preeclampsia compared to women without this condition [53]. To date, a substantial corpus of data has been accumulated on the changes in EV content observed in pregnancies affected by preeclampsia. A comprehensive overview of the principal studies examining the clinical utility of EVs in preeclampsia is presented in Table 1.

**Table 1:** The clinical values of EVs in preeclampsia.

Year Ref.	Extracellular Vesicles	Source of EVs	Groups	Pregnancy Stage	Method	Main Findings
2013 [9]	EVs	Placental syncytiotrophoblast	PE (*n* = 11) Normal (*n* = 22)	30–42 weeks	Ultracentrifugation, differential centrifugation, WB	The total number of exosomes increases at PE
2013 [9]	EVs	Placental syncytiotrophoblast	PE (*n* = 11) Normal (*n* = 22)	30–42 weeks	Ultracentrifugation, differential centrifugation, WB	Higher levels of Flt-1/sFlt-1 isoforms in PE
2017 [12]	Exosomes	Plasma	PE (*n* = 15) Normal (*n* = 32)	11–14 weeks, 21–24 weeks, 31–34 weeks	Ultracentrifugation, differential centrifugation, ELISA	The concentration of exosomes is increased in PE regardless of gestational age, AUC = 0.745 ± 0.094 for all exosomes and 0.829 ± 0.077 for placental exosomes at 11–14 weeks gestation
2017 [12]	Exosomes	Plasma	PE (*n* = 15) Normal (*n* = 32)	11–14 weeks, 21–24 weeks, 31–34 weeks	Ultracentrifugation, differential centrifugation, NGS	The content of miR-486-1-5p, miR-486-2-5p in exosomes is increased in PE regardless of gestational age
2019 [47]	Exosomes	Serum	PE (*n* = 42) Normal (*n* = 39)	37–38 weeks	Commercial kit (Invitrogen, Carlsbad, CA, USA), qRT-PCR	Reduction in miR-548c-5p at PE
2017 [52]	Exosomes	Plasma	PE (*n* = 23) Chronic hypertension (*n* = 16) Gestational hypertension (*n* = 14) Normal (*n* = 34)	24–40 weeks	Commercial kit, qRT-PCR	The concentration of total miRNA is higher in PE (FC = 3.19, *p* < 0.001). The level of hsa-miR-210-3p is higher in PE
1998 [53]	Syncytiotrophoblast microparticles	Peripheral venous plasma, uterine venous plasma	PE (*n* = 20) Normal (*n* = 20) Nonpregnant females (*n* = 10)	232–233 days	Ultracentrifugation, FCM, time resolved fluoroimmunoassay	EV levels are elevated in the plasma of women with PE. EV levels are higher in plasma from uterine vessels compared to peripheral blood
2016 [54]	Exosomes	Plasma	Early onset-PE (<33 weeks) (*n* = 15) Late onset-PE (>34 weeks) (*n* = 15) Normal (<33 weeks) (*n* = 15) Normal (>34 weeks) (*n* = 15)	26–39 weeks	Ultracentrifugation, differential centrifugation, ELISA	The total number of exosomes increases in PE regardless of the time of manifestation. The concentration of placental exosomes increases in early PE but decreases in late PE
2019 [55]	Exosomes	Plasma	Early onset-PE (<33 weeks) (*n* = 15) Late onset-PE (>34 weeks) (*n* = 15) Normal (<33 weeks) (*n* = 15) Normal (>34 weeks) (*n* = 15)	26–39 weeks	Commercial kit miRCURY (Qiagen, Valencia, CA, USA), ELISA	The total number of exosomes is higher in PE regardless of gestational age. The concentration of placental exosomes is higher in early PE
2019 [54]	Exosomes	Plasma	Early onset-PE (<33 weeks) (*n* = 15) Late onset-PE (>34 weeks) (*n* = 15) Normal (<33 weeks) (*n* = 15) Normal (>34 weeks) (*n* = 15)	26–39 weeks	Commercial kit miRCURY (Qiagen, Valencia, CA, USA), NTA	59 miRNAs associated with early PE and 30 miRNAs associated with late PE were identified
2018 [56]	Exosomes	Plasma	PE (*n* = 100) Normal (*n* = 100)	23–25 weeks	Ultracentrifugation, qRT-PCR	The levels of miR-136, miR-494 and miR-495 are significantly higher at PE
2018 [56]	Exosomes	Umbilical cord mesenchymal stem cells	PE (*n* = 15) Normal (*n* = 15)	23–25 weeks	Ultracentrifugation, qRT-PCR	The levels of miR-136, miR-494 and miR-495 are significantly higher at PE
2018 [57]	Exosomes	Serum	PE (*n* = 10) Normal (*n* = 10)	36–40 weeks	Ultracentrifugation, differentialcentrifugation, qRT-PCR	Increased miR-155 content at PE
2019 [58]	Exosomes	Plasma	PE (*n* = 43) Gestational hypertension (*n* = 57) FGR (*n* = 63) Normal (*n* = 102)	10–13 weeks	Commercial kit miRCURY (Qiagen, Valencia, CA, USA), qRT-PCR	Low levels of miR-517-5p, miR-520a-5p and miR-525-5p in the first trimester are associated with subsequent pregnancy complications
2019 [59]	Exosomes, microvesicles	Plasma	PE (*n* = 14) Normal (*n* = 14)	33–40 weeks	Differential centrifugation, ultracentrifugation, immunoprecipitation, size exclusion chromatography and ultrafiltration, Neprilysin Activity Assay Kit (AnaSpec, Fremont, CA, USA), FCM, WB	In PE placental exosomes are increased and neprilysin expression is upregulated
2017 [60]	EVs	Urine	PE (*n* = 49) Normal (*n* = 42)	24 h before delivery	Without isolation, WB	The ratio of podocin-positive EVs to nephrin-positive EVs is significantly elevated at PE
2018 [61]	Exosomes	Urine	PE (*n* = 24) Normal (*n* = 24)	28–36 weeks	Differential centrifugation, WB	In PE NKCC2 and ENaC activity is increased and NCC activity is decreased
2014 [62]	EVs	Plasma	PE (*n* = 11) Normal (*n* = 11)	27–38 weeks	Biotinylation, Mass spectrometry, ELISA	In total, 87 proteins were detected in the CTB EVs population and 104 proteins in the AV EVs population at PE
2015 [63]	Exosomes	Umbilical cord blood	PE (*n* = 10) Normal (*n* = 10)	34–39 weeks	Ultracentrifugation, differential centrifugation, LC-MS	Differences were found in the content of 29 proteins between normal and PE. The level of 14 proteins is increased and 15 is decreased in PE
2016 [64]	Exosomes	Gingival crevicular fluid	PE (*n* = 10) Normal (*n* = 20)	33–34 weeks	Ultracentrifugation, differential centrifugation, ELISA	The ratio of placental exosomes to their total number is significantly higher in patients with PE
2016 [64]	Exosomes	Gingival crevicular fluid	PE (*n* = 10) Normal (*n* = 20)	33–34 weeks	Ultracentrifugation, differential centrifugation, ELISA	PLAP concentrations are elevated in patients with PE
2016 [64]	Exosomes	Saliva, gingival crevicular fluid	PE (*n* = 10) Normal (*n* = 20)	33–34 weeks	Ultracentrifugation, differential centrifugation, ELISA	The concentration of sFlt-1 is significantly higher in patients with PE
2016 [65]	Apoptotic bodies, microvesicles, exosomes	Plasma	PE (*n* = 19) Normal (*n* = 14)	31–39 weeks	Ultracentrifugation, differential centrifugation, qRT-PCR	miR-885-5p, which is elevated in the plasma of pregnant women with PE, predominantly accumulates in plasma exosomes in PE (32-fold higher content compared to apoptotic cells)
2018 [66]	Exosomes	Plasma	PE (*n* = 10) Normal (*n* = 10)	36–38 weeks	Precipitation (commercial kit), Transmission Electron Microscopy, NTA, ELISA	Exosomes obtained from patients with PE contain significantly elevated concentrations of sFlt1 and sEng, contributing to endothelial vasoconstrictor dysfunction
2019 [67]	Placental EVs	Plasma	PE (*n* = 10) Normal (*n* = 7)	33 weeks	Not mentioned, FCM	The number of placental EVs is significantly higher at PE
2020 [68]	Placental exosomes	Plasma	PE (*n* = 21) Normal (*n* = 23)	31–33 weeks	Ultracentrifugation, differential centrifugation, NTA, WB	Syncytin-1 levels are decreased and PLAP levels are increased in patients with PE
2012 [69]	Syncytial aggregates	Plasma	PE (*n* = 16) Normal (*n* = 12)	28–40 weeks	Ultracentrifugation, differential centrifugation, WB	The concentration of sFlt1 is significantly higher at PE
2012 [69]	Syncytial aggregates	Placental villi	PE (*n* = 12) Normal (*n* = 9)	28–40 weeks	Ultracentrifugation, differential centrifugation, Immunohistochemistry, electron microscopy	Flt1/sFlt1 levels are significantly elevated at PE
2014 [70]	Microvesicles	Placental villi	PE (*n* = 3) Normal (*n* = 6)	>34 weeks, delivery	Differential centrifugation, LC-MS, immunohistochemistry	The content of 25 proteins differs in PE and normal pregnancy
2002 [71]	Microparticles	Plasma	PE (*n* = 16) Normal (*n* = 6)	27–31 weeks	Differential centrifugation, Wire myography	Microparticles derived from PE patients induce endothelial dysfunction in isolated myometrial arteries derived from healthy pregnant women
2006 [72]	Microparticles	Plasma	PE (*n* = 21) Normal (*n* = 17)	28–39 weeks	Differential centrifugation, Phenotyping using specific monoclonal antibodies	The total number of microparticles is significantly increased in PE. Leukocyte- and platelet-derived microparticles are also increased in PE
2007 [73]	Microparticles	Plasma	PE (*n* = 10) Normal (*n* = 10)	27–32 weeks	Differential centrifugation, FCM	No differences in complement system activation were found in healthy pregnant women and patients with PE
2008 [74]	Microparticles	Plasma	PE (*n* = 11) Normal (*n* = 8)	12–38 weeks	Differential centrifugation, FCM	In PE, in the third trimester, the total number of microparticles decreases, but placental, monocytic, and erythrocytic microparticles are increased
2009 [75]	Microparticles	Plasma	PE (*n* = 10) Normal (*n* = 10) Nonpregnant females (*n* = 10)	27–33 weeks	Differential centrifugation, FCM, ELISA, MLPA	The total number of microparticles is reduced in PE. The concentration of sl-selectin is significantly decreased and the concentration of elastase is significantly increased in patients with PE
2012 [76]	Microparticles	Plasma	PE (*n* = 8) Normal (*n* = 8)	27–34 weeks	Differential centrifugation, FCM	ICAM-1 content was significantly increased in monocyte cocultures and endothelial cells after addition of isolated microparticles from PE patients
2012 [77]	Microparticles	Plasma	Early-onset PE (<34 weeks)(*n* = 15) Late-onset PE (>34 v)(*n* = 15) Normal (*n* = 10)	28–38 weeks	Differential centrifugation, ELISA, WB	The concentration of placental microparticles is significantly higher in the PE group with early-onset. Caspase-3 levels are elevated in groups with PE
2004 [78]	Endothelial EVs < 1 µm	Plasma	PE (*n* = 52) Gestational hypertension (*n* = 20) Normal (*n* = 38)	>34 weeks	Ultrafiltration, FCM	Increased concentration of CD31+/42b- and CD62E+ BBs in women with PE and HG compared to controls, increased concentration of CD31+/42b- and CD62E+- BBs in women with PE compared to GH
2006 [79]	Microparticles	Plasma	Early-onset PE (*n* = 15) Late-onset PE (*n* = 10) Normal (*n* = 35)	24–40 weeks	Ultracentrifugation, ELISA	Microparticle levels are elevated in PE with early onset compared to normal pregnancy
2012 [80]	Microparticles	Plasma	PE (*n* = 58) Normal (*n* = 38) FGR(*n* = 12)	24–41 weeks	Differential centrifugation, FCM	Endothelial microparticle content was significantly increased in PE as in the PE + FGR group
2012 [81]	Microparticles of neutrophils, endothelial cells, monocytes, platelets, leukocytes, erythrocytes and syncytiotrophoblast	Plasma	PE (*n* = 28) Normal (*n* = 30)	30–36 weeks	Differential centrifugation, FCM	PE has significantly elevated levels of microparticles from a variety of sources
2012 [82]	Endothelial microparticles	Plasma	PE (*n* = 20) Normal (*n* = 20)	>36 weeks	Differential centrifugation, ELISA, FCM	Concentrations of all endothelial microparticles (CD31+/42-, CD 62E+ and CD105+) are significantly higher in patients with PE. The content of sFlt1 and sEnd is significantly elevated and PlGF is significantly decreased in PE
2014 [83]	Leukocyte microparticles	Plasma	PE (*n* = 24) Normal (*n* = 20)	38–39 weeks	Differential centrifugation, Cytofluorometry	The concentration of leukocyte microparticles does not differ between PE and control groups. In PE, the content of CD45 + CD16 − CD56 + microparticles is higher
2015 [84]	Microparticles	Plasma, cord blood	PE (*n* = 16) Normal (*n* = 16)	37–39 weeks	Differential centrifugation, FCM	In plasma, the total microparticle content as well as the concentration of leukoic microparticles is higher in PE. The concentration of platelet microparticles (CD61+) is lower in PE. No differences in the content of endothelial microparticles were detected. In cord blood samples, the content of all detected microparticles is significantly higher in PE
2016 [85]	Platelets and endothelial microparticles	Plasma	PE (*n* = 20) Normal (*n* = 20) Nonpregnant females (*n* = 20)	38–39 weeks	Differential centrifugation, FCM	Microparticles from women with PE decreased CD18 expression on the tumour necrosis factor α (TNF-α)-activated monocyte cell line TNR-1. Microparticles from healthy pregnant women increased the expression of CD18, CD54 and integrin β7 and decreased the expression of CD11a and CD29. Microparticles from nonpregnant women decreased the expression of CD18, CD49d and CD54 and increased the expression of CD11c, CD31, CD47 and vascular endothelial growth factor receptor 2
2015 [86]	Platelet and endothelial microparticles	Plasma	Severe PE (*n* = 35) Mild PE (*n* = 40) Normal (*n* = 60)	>34 weeks	Differential centrifugation, FCM	The concentration of endothelial microparticles is significantly higher in the group with severe PE; the concentration of trophoblast microparticles has no significant difference in the groups
2017 [87]	Microvesicles and exosomes of syncytiotrophoblast	Plasma, placenta	Plasma: PE (*n* = 6) Normal (*n* = 6) Placenta: PE (*n* = 8) Norma (*n* = 11)	28–40 weeks	Ultracentrifugation, differential centrifugation, FCM, WB	In PE, there is a decrease in eNOS levels in both plasma and placental EVs
2019 [88]	Exosomes	Urine	PE (*n* = 29) Normal (*n* = 23) Nonpregnant females (*n* = 19)	28–40 weeks	Differential centrifugation, WB	PFKFB2 levels were increased 4.7-fold in PE compared to controls (*p* < 0.001)
2017 [89]	Microparticles	Placental trophoblast	PE (*n* = 6) Normal (*n* = 25)	35–40 weeks, delivery	Differential centrifugation, FCM	Trophoblasts obtained from PE patients secrete significantly more microparticles (*p* < 0.01)
2019 [90]	Exosomes	Plasma, placenta	Plasma: PE (*n* = 8) Normal (*n* = 8) Placenta: PE (*n* = 13) Normal (*n* = 7)	28–40 weeks, delivery	Membrane affinity spin column (Qiagen, Valencia, CA, USA), qRT-PCR	The level of hsa-miR-210 is significantly elevated in samples with PE
2017 [91]	Microparticles	Plasma	PE (*n* = 33) Normal (*n* = 112)	10–14 weeks	Differential centrifugation, ELISA	In patients with PE, total annexin V and copeptin levels were significantly elevated, while PlGF levels, on the contrary, were significantly decreased compared to controls

PE, preeclampsia; FGR, foetal growth restriction; ELISA, enzyme-linked immunosorbent assay; NGS, next generation sequencing; NTA, nanoparticle tracking analysis; qRT-PCR, quantitative polymerase chain reaction; MLPA, multiplex ligation-dependent probe amplification; FCM, flow cytometry; WB, Western blotting; LC-MS, liquid chromatograph-mass spectrometer; sFlt1, soluble Fms-like tyrosine kinase-1; sEng, soluble endoglin; PlGF, placental growth factor; SWATH-MS, sequential windowed acquisition of all theoretical mass spectra.

The studies differ in terms of the type of EVs analysed, the biological material used, the characteristics of the groups under consideration, and the methods employed for the isolation and analysis of EVs. In the majority of studies, biological material was obtained non-invasively. In 35 out of 50 studies, the material was blood plasma, in three studies it was urine, in two studies it was serum, and in one study it was saliva. The number of patients included in the studies ranged from 3 to 100, while the control groups ranged from 6 to 112. The majority of studies were conducted during the second and third trimesters of pregnancy, with two studies examining the first trimester [58,91]. Two studies encompassed the entire gestational period [12,74].

The findings indicate an elevation in the overall plasma concentration of EVs in women diagnosed with preeclampsia (PE) during the third trimester [12,79,81,82,83,85]. Additionally, an increase in the total number of exosomes in the plasma of women with PE has been demonstrated during the third trimester [9,12,55]. It is noteworthy that the concentration of PEVs is elevated in the plasma of patients with PE in the third trimester [67]. Chaparro et al. demonstrated that the ratio of placental exosomes to the total number of exosomes in gingival crevicular fluid samples was significantly higher in patients with PE in the third trimester [64]. Some evidence indicates that the concentration of endothelial EVs is elevated in the third trimester in women with severe PE [83,87].

Additionally, the literature contains conflicting data. Three studies reported a decrease in circulating EVs levels in PE compared to healthy controls in the third trimester [54,74,75]. These discrepancies may be explained by different study designs, EV types, sources of EV, subtypes of PE, and other factors. Lok et al. demonstrated a reduction in the overall number of microparticles, but an increase in those derived from the placenta, monocytes and erythrocytes during the third trimester of pregnancies affected by PE [75]. Some studies have reported higher concentrations of total exosomes significantly in early onset-PE and late onset-PE, but the relative concentration of placental-derived exosomes significantly increased in early onset-PE and decreased in late onset-PE [54,55,77,79]. These facts suggest that early-onset and late-onset pre-eclampsia may have different etiologies and pathogenesis.

A number of studies have demonstrated that the plasma concentration of all exosomes and the concentration of placental exosomes are elevated throughout the course of pregnancy [12,55]. Specifically, the concentration of exosomes is elevated at 11–14 weeks prior to the onset of PE symptoms (AUC = 0.745 ± 0.094 for all exosomes, and AUC = 0.829 ± 0.077 for placental exosomes) [12].

Furthermore, the specific protein composition of EVs in pregnant women with PE was examined, in addition to the alterations in EV concentration. It has been demonstrated that PE is associated with an increase in the concentration of foetal HLA-G-labelled EVs in the blood of pregnant women [34]. Baig et al. identified 25 proteins in EVs that are associated with PE, including annexins, integrins, histones, heat shock proteins, complement regulation proteins, cytoskeleton proteins and various enzymes [70]. Histones, integrins and CD59 glycoprotein have been demonstrated to be reduced in concentration, while the levels of other proteins are elevated in PEVs [70]. These findings lend support to the hypothesis that PEVs play a role in the pro-inflammatory state observed in PE [70].

Endoglin (Eng) and Fms-like tyrosine kinase-1 (Flt-1), as well as their soluble forms (sEng and sFlt-1), have been observed to be elevated in placental exosomes and microvesicles of women with PE [9,64,69,83]. Flt-1 and Eng, as well as their soluble forms, have been demonstrated to reduce the bioavailability of the proangiogenic markers vascular endothelial growth factor (VEGF) and placental growth factor (PlGF), thereby promoting vasoconstriction, endothelial dysfunction and PE [66,69,74]. In vitro, exosomes derived from PE patients containing elevated concentrations of sFlt1 and sEng have been observed to attenuate the proliferation, migration, and tube formation of human umbilical vein endothelial cells [66].

The level of endothelial nitric oxide synthase (eNOS), which synthesises nitric oxide (NO), a vasodilator with beneficial effects on endothelial function during pregnancy, is reduced in placental-derived extracellular vesicles (PEVs) from patients with preeclampsia (PE) [87]. A reduction in eNOS levels results in a decline in NO production, which in turn contributes to endothelial vascular dysfunction and, subsequently, the development of arterial hypertension and proteinuria [87].

In the context of physical education, the levels of neprilysin, a membrane-bound metalloprotease with the capacity to reduce the activation of peptides including vasodilators, natriuretics and diuretics, are observed to be elevated in the PEVs [59]. The elevated expression of neprilysin is postulated to contribute to the hypertension and amyloid deposition that frequently accompanies PE [59].

A proteomic analysis of exosomes obtained from cord blood revealed that the levels of 14 proteins were elevated while 15 were diminished in pregnancies affected by PE [63]. These proteins are involved in a range of functions, including enzymatic regulatory activities, biological regulation and cellular processes [63].

A total of nine studies have analysed the miRNAs contained in EVs [12,47,52,55,56,57,58,65,90]. In one study, next-generation sequencing (NGS) was employed to demonstrate that the content of exosomal miR-486-1-5p and miR-486-2-5p was markedly elevated in preeclampsia (PE), irrespective of gestational age. This finding suggests that these miRNAs may serve as potential biomarkers for the early diagnosis of PE [12]. Other studies have employed qPCR [47,55,56,57,58,65].

Biro et al. observed that the concentration of total miRNA, as well as the level of miR-210-3p, was elevated in pregnancies affected by PE compared to healthy pregnancies [52]. miR-210 is a hypoxia marker, and an increased level of this miRNA may indicate inadequate blood supply to placental tissues [92]. It has been demonstrated that miR-210 plays a pivotal role in the aetiology of PE [50,91,93,94]. It has been demonstrated that miR-210 impairs the processes of proliferation, migration and invasion of trophoblast cells [95]. Experimental evidence has demonstrated that miR-210 suppresses the gene expression of the enzyme hydroxysteroid (17β) dehydrogenase (HSD17B1), which is synthesised by the placenta and responsible for the conversion of estrone to 17β-estradiol [96]. A reduction in the levels of this enzyme has been linked to uteroplacental and systemic vasoconstriction, which can result in the onset of endothelial dysfunction and the development of PE [96]. Consequently, miR-210 suppresses the expression of numerous genes that regulate processes such as migration, trophoblast invasion, angiogenesis, and endothelial functions, thereby playing a pivotal role in the pathogenesis of PE [50].

The level of miR-155 is elevated in serum-derived placental exosomes in patients with PE compared to the control group [57]. The analysis of miRNAs from serum samples of women who later developed PE revealed an increase in miR-155 and a decrease in three miRNAs (miR-26b-5p, miR-7-5p and miR-181a-5p) that are associated with hypertension [96]. Previous studies have demonstrated that miR-155 reduces the expression of endothelial nitric oxide synthase (eNOS) and the production of nitric oxide (NO) in primary human umbilical vein endothelial cells (HUVECs) [57].

The levels of miR-136, miR-494 and miR-495 are markedly elevated in exosomes derived from the peripheral blood of pregnant patients with PE in comparison to those of normal pregnancies [56]. These miRNAs may contribute to the aetiology of PE by inhibiting cell proliferation and apoptosis [56]. The elevated expression of miR-136, miR-494, and miR-495 in the early stages of pregnancy is associated with the development of PE [56]. Consequently, these miRNAs have the potential to serve as non-invasive biomarkers for the early prediction of PE [56].

A significant reduction in miR-548c-5p levels was observed in serum exosomes of patients with PE in comparison to healthy pregnant women [47]. It is established that miR-548c-5p can inhibit the proliferation and activation of lipopolysaccharide-stimulated macrophages [47]. In PE, miR-548c-5p functions as an anti-inflammatory factor [47].

Pillay et al. conducted a comprehensive analysis of exosomal miRNAs in pregnant women with early-onset (≤33 weeks) and late-onset (≥34 weeks) PE [55]. Their findings revealed the presence of 59 miRNAs associated with early-onset PE and 30 miRNAs linked to late-onset PE [55]. The upregulation of miRNAs in PE is associated with the dysregulation of several biological processes, including cell migration and invasion, cell proliferation, apoptosis, mesenchymal transition and angiogenesis [55]. The levels of miR-2113 and miR-374c-5p were found to be significantly reduced in groups with early and late onset PE [55]. It is established that miR-2113 regulates fatty acid biosynthesis, which is a pivotal process in lipid metabolism, and that miR-374c regulates inflammation, which is a crucial biological process associated with the pathophysiology of PE [55].

The content of exosomal miR-486-1-5p and miR-486-2-5p is markedly elevated in pregnancies affected by PE, irrespective of gestational age. This observation renders these microRNAs promising biomarkers for the early diagnosis of PE [12].

According to the literature, there is an increase in different types of EVs in the biological fluids of pregnant women in cases of PE. It is postulated that alterations in the proteomic profile and miRNA composition of EVs may be the underlying cause of disorders of trophoblast invasion, angiogenesis, inflammatory response and endothelial dysfunction in PE. The obtained data substantiate the hypothesis that EVs play a pathogenic role in the development of PE. The potential for utilising biomolecules transported within EVs for diagnostic purposes to anticipate the likelihood of PE has been substantiated.

### 4.2. Gestational Diabetes Mellitus

Gestational diabetes mellitus (GDM) is a pathological condition characterised by hyperglycaemia, initially identified during pregnancy but not meeting the criteria for “manifest” diabetes mellitus (World Health Organization, 2014 [97]). The global incidence of GDM is estimated to be approximately 14% of pregnancies, representing approximately 20 million births annually [98]. GDM can cause adverse pregnancy outcomes, such as PE, PTB and foetal macrosomia, and is also associated with a high risk of developing distant complications such as type 2 diabetes mellitus and metabolic syndrome in both the mother and the foetus [99]. The precise causes and mechanisms of hyperglycaemia in pregnancy remain unclear [100]. There is an increasing body of evidence that EVs may play a significant role in the aetiology and pathogenesis of GDM [101,102]. The principal studies that substantiate this assertion are presented in Table 2.

**Table 2:** The clinical values of EVs in gestational diabetes mellitus.

Year Ref.	Extracellular Vesicles	Source of EVs	Groups	Pregnancy Stage	Method	Main Findings
2020 [40]	Placental exosomes	Urine	GDM (*n* = 27) Normal (*n* = 34)	8–39 weeks	Centrifugation, qRT-PCR	In the second trimester of pregnancy, increased expression of miR-516-5p, miR-517-3p, miR-518-5p, miR-222-3p and miR-16-5p was observed in GDM patients
2018 [101]	Exosomes	Plasma, chorionic villi	GDM (*n* = 12) Normal (*n* = 12)	37–39 weeks	Ultracentrifugation, differential centrifugation, NGS, qRT-PCR	In GDM, 9 miRNAs are upregulated and 14 miRNAs are downregulated in exosomes of placental origin. The content of miRNAs hsa-miR-125a-3p, hsa334 miR-99b-5p, hsa-miR-197-3p, hsa-miR-22-3p and hsa-miR-224-5p is the same in exosomes derived from plasma and chorionic villi during GDM
2016 [103]	Exosomes, microvesicles	Plasma	GDM (*n* = 7) Normal (*n* = 13)	11–14 weeks, 22–24 weeks, 32–36 weeks	Ultracentrifugation, differential centrifugation, ELISA	GDM doubly increases the concentration of exosomes. Exosomes from early, mid, and late gestation obtained from normal pregnancy significantly increased (∼1.8-fold) the release of GM-CSF, IL-4, IL-6, IL-8, IFN-γ, and TNF-α, without significant differences between gestational age
2019 [104]	EVs	Gingival crevicular fluid	GDM (*n* = 11) Normal (*n* = 23)	11–14 weeks	Commercial kit ExoQuick (System Biosciences Inc., Mountain View, CA, USA), NTA, ELISA, transmission electron microscopy	The concentration of EVs is significantly higher with GDM (AUC = 0.81)
2019 [105]	EVs	Serum	GDM (*n* = 23) Normal (*n* = 46)	6–15 weeks	Ultracentrifugation, differential centrifugation, qRT-PCR	The level of 10 miRNAs (miR-520h, miR-1323, miR-136-5p, miR-342-3p, miR-29a-3p, miR-29b-3p, miR-122-5p, miR-132-3p. miR-182-3p and miR-210-3p) were significantly higher in the group with GDM
2019 [106]	Placental EVs	Plasma	GDM (*n* = 6) Normal (*n* = 6)	39–40 weeks	Ultracentrifugation, differential centrifugation, FCM	The amount of dipeptidyl peptidase IV (DPPIV) is increased with GDM
2019 [107]	EVs	Plasma	GDM (*n* = 6) Normal (*n* = 19)	6–36 weeks	Differential centrifugation, NTA	The concentration of extracellular vesicles collected in the first trimester was significantly higher in patients who subsequently developed GDM
2020 [108]	EVs	Plasma	GDM (*n* = 24) Normal (*n* = 24)	24–28 weeks	Differential centrifugation, NTA	The number of EVs of placental origin is significantly higher in GDM (higher PLAP level)
2021 [109]	EVs	Plasma	GDM (*n* = 50) Normal (*n* = 50)	28–40 weeks	Not mentioned, FCM	The total number of EVs is slightly higher in the group with GDM, and the number of adipocytic EVs is higher in the control group. A significant correlation between the percentage of adipocytic EVs and total cholesterol is observed in the GDM group
2020 [110]	Exosomes	Umbilical cord blood	GDM (*n* = 23) Normal (*n* = 23)	Delivery	Ultracentrifugation, differential centrifugation, qRT-PCR	The concentration of exosomes was significantly higher in GDM patients. We identified 507 differentially expressed circular RNAs, of which 229 were activated and 278 were suppressed in patients with GDM
2019 [111]	Exosomes	Adipose tissue	GDM (*n* = 82) Normal (*n* = 65)	>37 weeks (delivery)	Differential centrifugation, exclusion chromatography, NTA	The number of exosomes is significantly higher in GDM. We identified 127 proteins whose expression is statistically significantly altered in GDM, of which 110 proteins are activated and 17 are repressed
2016 [112]	Exosomes	Urine	GDM (*n* = 8) Normal (*n* = 10)	20 weeks	Differential centrifugation, LC-MS, WB	Identified 645 proteins in exosomes of healthy pregnant women and 855 proteins in exosomes of GDM patients. The content of 70 proteins differed significantly between the compared groups, with the most significant differences found for the calcium-binding protein S100 A9 (S100A9)
2021 [113]	Exosomes	Cord blood, umbilical cord, placenta.	GDM (*n* = 23) Normal (*n* = 47)	Delivery (38–39 weeks)	Commercial kit Exoquick™ Exosome Precipitation Solution (System Biosciences, Mountain View, CA, USA), qRT-PCR	A statistically significant increase in miR-126-3p was observed in all types of biomaterial examined
2022 [114]	EVs	Plasma	GDM (*n* = 20) Normal (*n* = 25)	11–14 weeks	Centrifugation, NTA	The concentration of EVs was significantly higher in patients with subsequently developed GDM (AUC = 0.813 ± 0.080)

GDM, gestational diabetes mellitus; ELISA, enzyme-linked immunosorbent assay; NGS, next generation sequencing; NTA, nanoparticle tracking analysis; qRT-PCR, quantitative polymerase chain reaction; FCM, flow cytometry; WB, Western blotting; LC-MS, liquid chromatograph-mass spectrometer; GM-CSF, granulocyte-macrophage colony-stimulating factor; IFN-γ, Interferon gamma; TNF-α, Tumor Necrosis Factor α.

The presented studies exhibit notable differences in terms of the type of EVs analysed, the biological material employed, gestational age and the basic methods utilised. The biological material employed in the analysis of PEVs included blood plasma, urine, serum, gingival fluid, adipose tissue, cord blood and placenta. The number of patients diagnosed with GDM ranged from 6 to 82, while the comparison group ranged from 6 to 65. Three studies were conducted during the first trimester of pregnancy [103,104,111]. Four studies conducted analyses throughout the entire gestational period [40,102,106,108]. The content of EVs was examined in five studies at both the third trimester of pregnancy and at the time of delivery [100,105,109,110,112]. Notwithstanding the disparate study designs, analogous results were yielded. The presence of GDM was associated with elevated levels of total and placental exosomes throughout the gestational period [102,109,110]. Additionally, an increase in the number of PEVs has been observed in individuals with GDM [107]. Furthermore, an elevation in plasma EV concentration has been documented as early as the first trimester of pregnancy, preceding the onset of clinical manifestations associated with GDM. In a study conducted by Arias et al., elevated concentrations of EVs were observed in plasma samples obtained during the first trimester from women who subsequently developed GDM [107]. Additionally, the work of Russian scientists demonstrated a considerable elevation in the plasma concentration of EVs during the first trimester in women who subsequently developed GDM [114]. The area under the ROC curve was 0.813 ± 0.080, and the sensitivity and specificity of the model were 80.0% and 66.7%, respectively [114]. It has been demonstrated that in women with GDM, the concentration of EVs is elevated not only in the plasma, but also in other biological fluids. Therefore, Monteiro et al. demonstrated that at 11–14 weeks of gestation, the concentration of EVs obtained from gingival fluid is elevated in women who were subsequently diagnosed with GDM (AUC = 0.81) [104].

A growing body of evidence suggests that EVs may play a pivotal role in glucose metabolism and the regulation of the insulin response. EVs isolated from the plasma of “conditionally healthy” pregnant women in non-pregnant mice have been observed to stimulate insulin production by β-cells in response to glucose and to increase insulin sensitivity [108]. Conversely, EVs obtained from women with GDM have been observed to decrease insulin production and cause increased insulin resistance [108]. Exosomes derived from the placenta and isolated from the plasma of women with GDM have been observed to reduce glucose transport and uptake by muscle cells from donors without impaired insulin sensitivity [101]. Conversely, placental exosomes derived from pregnant women without impaired glucose tolerance have been demonstrated to enhance glucose uptake by muscle cells from diabetic patients [101].

Furthermore, there is evidence to suggest that EVs may have a detrimental impact on the progression of GDM. For example, exosomes isolated from the plasma of women with GDM have been demonstrated to significantly increase the release of pro-inflammatory cytokines (GM-CSF, IL-4, IL-6, IL-8, IFN-γ and TNF-α) from endothelial cells [103], which may serve to exacerbate the course of GDM. This is due to the fact that excessive inflammation contributes to insulin resistance and hence impaired glucose metabolism, as well as the development of additional pregnancy complications.

A differential proteomic composition of EVs has been identified in patients with GDM and women without pregnancy complications. In their examination of plasma PEVs, Jayabalan and colleagues identified differences in the content of 78 proteins [102]. Of particular interest are calcium/calmodulin-dependent protein kinase II beta (CAMK2β) and pappalisin-1 (PAPP-A), which affect insulin signalling and glucose metabolism [102]. Kandzija et al. observed an increase in dipeptidyl peptidase IV (DPPIV), which cleaves glucagon-like polypeptide-1 (GLP-1), in the PEVs of women with GDM [106]. The latter is produced in response to food intake and stimulates insulin secretion [106]. A reduction in GLP-1 levels in GDM may result in a corresponding reduction in insulin secretion, leading to hyperglycaemia [106]. A proteomic analysis of exosomes from urine identified 70 proteins associated with GDM, with the most significant differences observed for the calcium-binding protein S100A9, which has been identified as a marker of inflammation [112]. Furthermore, the level of S100A9 protein has been demonstrated to correlate with obesity in pregnant women and macrosomia in the foetus [112]. The examination of exosomes derived from adipose tissue of patients with GDM yielded intriguing results [111]. A total of 110 proteins were identified as being elevated, while 17 proteins were found to be decreased [111]. These proteins are involved in the regulation of mitochondrial dysfunction, the sirtuin signalling pathway, the mechanistic target of rapamycin (mTOR) signalling pathway, and oxidative phosphorylation [111]. The authors concluded that exosomes derived from adipose tissue of patients with GDM exert an influence on the processes of glucogenesis in the placenta [111]. 

The RNA content of EVs in plasma, urine, placenta, umbilical cord and cord blood was determined by qPCR. Therefore, in GDM, the level of 9 miRNAs is increased and that of 14 miRNAs is decreased in exosomes of placental origin [101]. In women with GDM, exosomes derived from maternal blood plasma and chorionic villi exhibited comparable levels of miR-125a-3p, hsa-334, miR-99b-5p, miR-197-3p, miR-22-3p and miR-224-5p miRNAs [101]. Gillet et al. conducted an analysis of the miRNA profile contained in serum EVs during the early stages of pregnancy (6–15 weeks) [105]. Their findings revealed the presence of 10 miRNAs (miR-520h, miR-1323, miR-136-5p, miR-342-3p, miR-29a-3p, miR-29b-3p, miR-122-5p, miR-132-3p, miR-182-3p and miR-210-3p) with elevated levels in women with GDM [105]. A bioinformatics analysis has demonstrated that these miRNAs are implicated in trophoblast proliferation and differentiation, as well as in insulin regulation and glucose transport during pregnancy [105]. A change in the content of miRNAs in placental exosomes isolated from the urine of patients with GDM is observed throughout the course of pregnancy [40]. Herrera-Van Oostdam et al. identified miRNAs that have the greatest potential as biomarkers of GDM (miR-516-5p, miR-517-3p, miR-518-5p, miR-222-3p and miR-16-5p), whose levels are elevated in the second trimester of pregnancy in patients with GDM [40]. These miRNAs regulate the activity of genes involved in the regulation of various metabolic, molecular and cellular processes, the most important of which are fatty acid biosynthesis, the PI3K-Akt signalling pathway and the insulin signalling pathway [40]. An examination of the contents of exosomes obtained from placenta, umbilical cord and cord blood revealed an increase in miR-126-3p in all types of samples from patients with GDM [113]. This miRNA is involved in the regulation of angiogenesis and the inflammatory response [113]. An analysis of the profile of circular RNAs in cord blood exosomes revealed that the level of 229 RNAs is increased and 278 is decreased in patients with GDM [110]. These RNAs are involved in the regulation of galactose metabolism, glycan biosynthesis, cholesterol metabolism, DNA replication and RNA transport, which are related to glycometabolism and lipometabolism. The disruption of these processes is associated with GDM [110].

Consequently, alterations in the concentration of EVs in blood plasma are linked to the progression and clinical trajectory of GDM. The stimulation of immune and inflammatory reactions in patients with GDM by EVs determines a more severe clinical course. Concurrently, the evaluation of EV concentration and the examination of their contents during pregnancy represent a promising avenue for the prediction and monitoring of GDM. Of particular interest are miRNAs (miR-520h, miR-1323, miR-136-5p, miR-342-3p, miR-29a-3p, miR-29b-3p, miR-122-5p, miR-132-3p, miR-182-3p and miR-210-3p), whose elevated levels in the first trimester may indicate the subsequent development of GDM.

### 4.3. Foetal Growth Restriction

FGR represents one of the primary causes of stillbirth, neonatal mortality, and postnatal morbidity [115]. FGR is defined as foetal growth retardation resulting in a deviation from the estimated gestational age and birth weight below the 10th percentile for the gestational age, as determined by linear measurements [115]. The prevalence of FGR is estimated to be between 5 and 18% [2]. The underlying pathogenesis of FGR is believed to be associated with placental dysfunction, although the specific causes remain unclear [115]. The link between alterations in the concentration of circulating EVs and their contents and the emergence of FGR has been demonstrated in numerous studies, as outlined in Table 3.

**Table 3:** The clinical values of EVs in foetal growth restriction.

Year Ref.	Extracellular Vesicles	Source of EVs	Groups	Pregnancy Stage	Method	Main Findings
2019 [58]	Exosomes	Plasma	PE (*n* = 43) Gestational hypertension (*n* = 57) FGR (*n* = 63) Normal (*n* = 102)	10–13 weeks	Commercial kit miRCURY (Qiagen, Valencia, CA, USA), qRT-PCR	Low levels of miR-517-5p, miR-520a-5p and miR-525-5p in the first trimester are associated with subsequent pregnancy complications
2012 [80]	Microparticles	Plasma	PE (*n* = 58) Normal (*n* = 38) FGR (*n* = 12)	24–41 weeks	Differential centrifugation, FCM	The content of microvesicles is lower in the PE + FGR group than in the group with PE. The number of microvesicles in the blood of pregnant women with FGR does not change
2018 [116]	Exosomes	Plasma	FGR (*n* = 20) Normal (*n* = 10)	33–37 weeks	Ultracentrifugation, differential centrifugation, ELISA, NTA	In foetal growth retardation, the number of placental exosomes is significantly reduced
2017 [117]	Exosomes	Serum	FGR (*n* = 36) Normal (*n* = 51)	16–22 weeks	Ultracentrifugation, differential centrifugation, qRT-PCR	High levels of miR-20b-5p, miR-942-5p, miR-324-3p, miR-223-5p, and miR-127-3p are associated with a low risk of foetal growth retardation
2024 [118]	sEVs	Plasma	Small--for--gestational age (SGA) (*n* = 43) Normal (*n* = 220)	10–14 weeks, 16–22 weeks, 26–32 weeks, delivery	Ultracentrifugation, filtration, FCM, WB, NTA, lipid MS	The SGA group had lower EVs than controls throughout pregnancy. A lipid profile of 25 differentially expressed lipids was found to predict the birth of a small-for-gestational-age (SGA) infant in the first and second trimester of pregnancy (AUC = 0.822 and 0.909, respectively)
2022 [119]	Microparticles	Plasma	FGR (*n* = 32) Normal (*n* = 20)	24 weeks– delivery	Differential centrifugation, WB	In the third trimester, OPA1 protein content was significantly increased in the FGR group compared to controls

sEVs, small extracellular vesicles; PE, preeclampsia; FGR, foetal growth restriction; ELISA, enzyme-linked immunosorbent assay; NGS, next generation sequencing; NTA, nanoparticle tracking analysis; qRT-PCR, quantitative polymerase chain reaction; FCM, flow cytometry; WB, Western blotting.

In their study, Miranda et al. analysed the number of total and placental exosomes in the plasma of women who had given birth to children with FGR using CD63 and PLAP markers [116]. The ratio of placental exosomes (PLAP + CD63+) to total exosomes (PLAP − CD63+) provides insight into the contribution of placental exosomes to the total number of exosomes [116]. A reduction in the concentration of placental exosomes has been demonstrated in cases of FGR [116]. Furthermore, the ratio of placental exosomes to the total number of exosomes has been demonstrated to correlate with foetal body weight, and thus may serve as a potential biomarker for FGR [116]. Conversely, Alijotas-Reig et al. observed no alteration in the quantity of microvesicles present in the blood of pregnant women with FGR [80].

A recent study demonstrated that the content of placental small extracellular vesicles (sEVs) was markedly reduced in a cohort of women with a foetus that was small for gestational age (SGA) in comparison to a control group of women with a foetus that was of the appropriate gestational age [118]. The lipid profile of placental sEVs was also analysed, which revealed 25 differentially expressed proteins in normal and SGA pregnancies [118]. Based on these data, a predictive model for SGA birth was proposed, with the area under the curve (AUC) values indicating that the greatest predictive effect is achieved in the first and second trimesters of pregnancy (AUC = 0.822 and 0.909, respectively) [118].

A team of Russian scientists has demonstrated that the content of key proteins involved in mitochondrial biogenesis undergoes changes during the second and third trimesters of pregnancy [119]. These proteins, which are responsible for maintaining mitochondrial functionality, preserving their integrity and regulating biogenesis (VDAC1, OPA1, DRP1 and TAZ), are present in the plasma microvesicles of the blood of patients with FGR. The findings indicated that mitochondrial biogenesis is impaired in pregnant women with FGR [119].

The analysis of miRNAs in plasma EVs demonstrated that elevated levels of miR-20b-5p, miR-942-5p, miR-324-3p, miR-223-5p and miR-127-3p in the second trimester of pregnancy were associated with a reduced risk of FGR [117]. The deletion of miR-127-3p in mice resulted in the development of a deficiency in the labyrinthine zone of the placenta, which is analogous to the villous zone of the human placenta, which is directly involved in the transfer of gases and nutrients between mother and foetus [120]. The data suggest that miR-127-3p may be associated with placental development and foetal growth. miR-942-5p and miR-20b-5p are involved in the regulation of angiogenesis and trophoblast development, which are essential for normal foetal growth and development [58,121].

A reduction in the level of miR-520a-5p in the first trimester is associated with the subsequent development of FGR [58]. The authors proposed that miR-520a-5p, which is highly expressed in the placenta and has a reduced expression level in other tissues, could be used as a biomarker of FGR [58].

It has been demonstrated that the concentration of placental EVs in the blood plasma of pregnant women is correlated with foetal body weight. The miRNAs present within EVs oversee the processes that are essential for the normal growth and development of the foetus. The data obtained suggest the potential for utilising EVs for foetal monitoring throughout the gestational period.

### 4.4. Preterm Birth

The term “preterm birth” is used to describe births that occur between 22 and 36 weeks and 6 days of gestation, as defined by the World Health Organization (WHO) [122]. According to the World Health Organization (WHO), approximately 15 million premature infants are born annually worldwide [122,123]. PTB represents a significant cause of mortality and morbidity among children under the age of five [122]. Recent studies have indicated that EVs may act as mediators in the initiation of labour [124]. A synthesis of the findings from studies examining the impact of EVs on the progression of PTB is presented in Table 4.

**Table 4:** The clinical values of EVs in preterm birth.

Year Ref.	Extracellular Vesicles	Source of EVs	Groups	Pregnancy Stage	Method	Main Findings
2019 [10]	Exosomes	Plasma	PTB (*n* = 10) Normal (*n* = 20)	9–40 weeks	Ultracentrifugation, differential centrifugation, NGS	The content of 173 miRNAs differs in PB and normal pregnancy
2019 [124]	Exosomes	Plasma	PTB (*n* = 13) Preterm premature rupture of membranes (*n* = 8) Term in labour (*n* = 11) Term not in labour (*n* = 13)	32–40 weeks	Ultracentrifugation, differential centrifugation, SWATH-MS	72 proteins were identified, the content of which differed between the study groups
2019 [125]	Microparticles	Plasma	PTB (*n* = 87) Normal (*n* = 174)	10–12 weeks	Exclusion chromatography, LC-MS	A set of proteins (F13A, FBLN1, IC1, ITIH2 and LCAT) showing high potential for use, as a PB biomarker, in early pregnancy was identified (AUC = 0.74 (95% DI, 0.63–0.81))
2018 [126]	EVs	Plasma	PTB (*n* = 20) Normal (*n* = 47)	24–34 weeks	Differential centrifugation, exclusion chromatography, NGS, qRT-PCR	A total of 535 microRNAs were identified, of which 51 had significant concentration changes between groups
2016 [127]	Bacterial EVs	Urine	PTB (*n* = 35) Normal (*n* = 39)	25–42 weeks	Ultracentrifugation, differential centrifugation, NGS	The level of bacterial EVs is higher at PB
2016 [128]	EVs	Plasma	PTB (*n* = 25) Normal (*n* = 50)	10–12 weeks	Exclusion chromatography, LC-MS	Identified 62 proteins that may be used as biomarkers of preterm birth
2015 [129]	Microparticles	Serum	PTB (*n* = 24) Normal (*n* = 24)	15–17 weeks	Differential centrifugation, LC	A total of 99 proteins were identified with statistically significant differences between the PB and control groups. An additional study identified 18 proteins associated with the risk of PB

PB, preterm birth; NGS, next generation sequencing; qRT-PCR, quantitative polymerase chain reaction; LC-MS, liquid chromatograph-mass spectrometer; SWATH-MS, sequential windowed acquisition of all theoretical mass spectra.

The studies are devoted to the analysis of EVs of different types in different biological material at different stages of pregnancy. In the majority of cases, blood plasma was employed as the biological material under investigation. The number of women who gave birth prematurely ranged from 10 to 87, with control groups ranging from 20 to 174. Three studies examined EVs during the first trimester of pregnancy [125,128,129]. One study examined EVs in the third trimester of pregnancy [124]. Another study examines the presence of EVs throughout the course of pregnancy [10]. Two papers present findings from the second and third trimesters of pregnancy [126,127].

The proteins present within EVs were subjected to analysis by mass spectrometry. A study examining the proteomic profile of plasma microparticles at 15 to 17 weeks gestation identified 18 proteins associated with the risk of PTB [129]. A significant proportion of these proteins are involved in antigen presentation and humoral immune and inflammatory pathways [129]. A proteomic analysis of EVs contents obtained at 10–12 weeks of gestation from women whose pregnancies subsequently ended in PTB at 34 weeks revealed 62 proteins associated with PTB and linked to inflammatory and coagulation processes [128]. A panel of three proteins, namely alpha-2-macroglobulin (A2MG), human endogenous ORF with a mean repetition rate of 34 (HEMO), and mannose-binding lectin 2 (MBL2), demonstrated a specificity of 83% with a mean AUC of 0.89, which can be employed as prognostic biomarkers of spontaneous PTB [128]. A comparable study compared the proteomic profile of exosomes derived from the plasma of women who gave birth at term, women with preterm birth (PTB), and non-pregnant women [124]. A total of 72 proteins were identified as being differentially expressed in the studied groups [124]. Bioinformatics analysis revealed that these proteins are associated with inflammatory and metabolic signals [124]. Proteins (F13A, FBLN1, IC1, ITIH2 and LCAT) with high diagnostic potential for PTB in early pregnancy (AUC = 0.74) have also been identified [125].

The concentration of bacterial EVs derived from *Ureaplasma* and *Veillonellaceae* is markedly elevated in the urine of women with PTB in comparison to women who have given birth at term [127].

The analysis of the profile of miRNAs isolated from serum exosomes identified 173 miRNAs that were found to be associated with PTB [10]. These include miR-155, miR-26b-5p, miR-181a-5p, miR-495 and miR-374c, whose expression is altered in PE [10,55,56,96]. Furthermore, alterations in the levels of miR-197-3p, miR-520h, miR-1323, miR-342-3p, miR-132-3p, miR-182-3p, miR-517-3p, miR-222-3p, miR-16-5p and miR-126-3p were also identified in GDM, as previously documented [10,55,101,105]. The level of miR-127-3p is altered in PTB and a low level is associated with a reduced risk of GDM [10,117,120]. A comparison of the profile of miRNAs contained in EVs from plasma revealed the presence of 51 miRNAs with significant concentration changes in preterm pregnant women [126].

A review of the literature reveals evidence of alterations in the composition of EVs in pregnancies affected by PTB. A number of biological molecules have been identified which demonstrate considerable potential for use in the screening and risk assessment of PTB in the early stages of pregnancy.

The presented data provide insight into the behaviour of EVs in normal and pathological pregnancy, indicating a potential for the development of new methods for early and effective diagnosis of pregnancy complications. Given their capacity to discern EVs and their contents in the peripheral blood of women from the earliest stages of pregnancy, they appear to be a promising avenue for the development of timely prognostic or diagnostic biomarkers for pregnancy complications and foetal abnormalities. Although there is evidence indicating a correlation between alterations in EV concentration and the emergence of pregnancy complications, it is premature to conclude that they can be employed as biomarkers. The absence of established, standardised methods for the detection and analysis of EVs has previously constituted an obstacle to the development of a sufficient evidence base for their introduction into clinical practice. However, in February 2024, the MISEV2024 guidelines were published, providing information for EVs research and outlining key methods and approaches for the isolation and analysis of EVs, which the society has reached consensus on [24]. The application of these methods and approaches will facilitate consensus among researchers regarding the results of studies examining the change in the number of EVs in different pregnancy pathologies and the diagnostic potential of this parameter. At this time, the most promising candidates for use as biomarkers appear to be the miRNAs carried by extracellular vesicles, as comparable results have been obtained for them in several studies. As demonstrated above, the expression of the majority of miRNAs linked to pregnancy complications undergoes alteration as early as the first trimester of pregnancy. This indicates that miRNAs extracted from the blood of a pregnant woman during the first trimester may serve as a potential indicator for the likelihood of complications. Such an early diagnosis will facilitate timely intervention, thereby reducing the risk of harm to the woman and the foetus.

## 5. Conclusions

**Figure 1:**
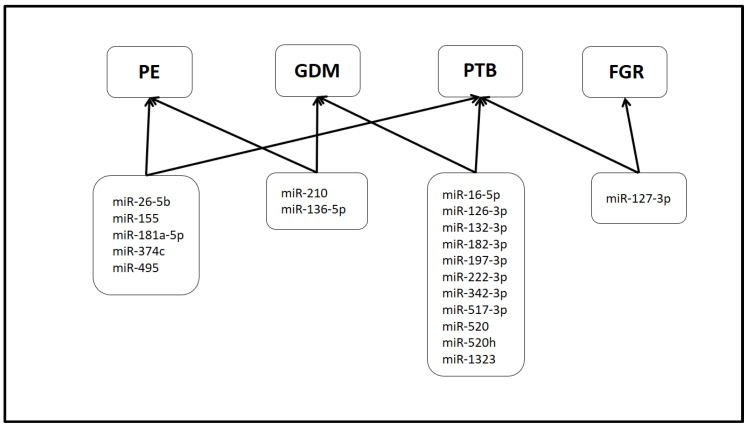
MiRNAs whose changes were detected in different pregnancy complications. PE—pre-eclampsia; GDM—gestational diabetes mellitus; PTB—preterm birth; FGR—foetal growth restriction.

It can be concluded that EVs play a role in the pathogenesis of pregnancy complications, including the provocation of systemic inflammation, disorders of glucose metabolism and angiogenesis regulation, trophoblast dysfunction and other pathological processes. The capacity to identify EVs in the blood of pregnant women at an early stage makes them a promising class of biomarkers for the timely prediction and diagnosis of pregnancy complications. Nevertheless, in order to assert that EVs can be employed as biomarkers of pregnancy complications, it is essential to amass a substantial body of evidence in which the findings of studies are consistent and reproducible. To date, studies have identified associations between changes in EV concentration and content and the development of pregnancy complications. The internal contents of EVs have been the subject of extensive study, although comparable results have been obtained for miRNAs. Consequently, elevated levels of miR-210 and miR-136-5p may serve as indicative markers for PE and GDM. The altered expression of miR-155, miR-26b-5p, miR-181a-5p, miR-495 and miR-374c is a characteristic feature of PE and PTB. Furthermore, altered expression of miR-197-3p, miR-520h, miR-1323, miR-342-3p, miR-132-3p, miR-182-3p, miR-517-3p, miR-222-3p, miR-16-5p and miR-126-3p has been linked to PTB and GDM. The level of miR-520 is elevated in women with GDM, and decreased levels of this microRNA are associated with PTB. Elevated levels of miR-127-3p are indicative of FGR and PTB. These findings are illustrated in a schematic diagram (Figure 1). Further large-scale studies and meta-analyses of EVs are warranted to gain a deeper understanding of the functions of EVs during pregnancy and to ascertain the potential use of EVs for the diagnosis, monitoring and treatment of pregnancy-related diseases. A significant breakthrough for clinical research and the use of EVs in medical practice would be the achievement of consensus on the adoption of EVs nomenclature and the validation of methods for isolating and analysing EVs and their contents.
